# TRPM4 Is a Novel Component of the Adhesome Required for Focal Adhesion Disassembly, Migration and Contractility

**DOI:** 10.1371/journal.pone.0130540

**Published:** 2015-06-25

**Authors:** Mónica Cáceres, Liliana Ortiz, Tatiana Recabarren, Anibal Romero, Alicia Colombo, Elías Leiva-Salcedo, Diego Varela, José Rivas, Ian Silva, Diego Morales, Camilo Campusano, Oscar Almarza, Felipe Simon, Hector Toledo, Kang-Sik Park, James S. Trimmer, Oscar Cerda

**Affiliations:** 1 Programa de Biología Celular y Molecular, Instituto de Ciencias Biomédicas (ICBM), Facultad de Medicina, Universidad de Chile, Santiago, Chile; 2 Department of Neurobiology, Physiology and Behavior, College of Biological Sciences, University of California Davis, Davis, California, United States of America; 3 Programa de Anatomía y Biología del Desarrollo, Instituto de Ciencias Biomédicas (ICBM), Facultad de Medicina, Universidad de Chile, Santiago, Chile; 4 Section on Cellular Signaling, Program in Developmental Biology, National Institute of Child Health and Human Development (NICHD), National Institute of Health, Bethesda, Maryland, United States of America; 5 Programa de Fisiopatología, Instituto de Ciencias Biomédicas (ICBM), Facultad de Medicina, Universidad de Chile, Santiago, Chile; 6 Departamento de Ciencias Biologicas, Facultad de Ciencias Biologicas, Universidad Andres Bello, Santiago, Chile; 7 Facultad de Medicina, Universidad Andres Bello, Santiago, Chile; 8 Millennium Institute on Immunology and Immunotherapy, Santiago, Chile; 9 Department of Physiology, School of Medicine, Kyung Hee University, Seoul, Korea; 10 Department of Physiology and Membrane Biology, School of Medicine, University of California Davis, Davis, California, United States of America; Seoul National University, REPUBLIC OF KOREA

## Abstract

Cellular migration and contractility are fundamental processes that are regulated by a variety of concerted mechanisms such as cytoskeleton rearrangements, focal adhesion turnover, and Ca^2+^ oscillations. TRPM4 is a Ca^2+^-activated non-selective cationic channel (Ca^2+^-NSCC) that conducts monovalent but not divalent cations. Here, we used a mass spectrometry-based proteomics approach to identify putative TRPM4-associated proteins. Interestingly, the largest group of these proteins has actin cytoskeleton-related functions, and among these nine are specifically annotated as focal adhesion-related proteins. Consistent with these results, we found that TRPM4 localizes to focal adhesions in cells from different cellular lineages. We show that suppression of TRPM4 in MEFs impacts turnover of focal adhesions, serum-induced Ca^2+^ influx, focal adhesion kinase (FAK) and Rac activities, and results in reduced cellular spreading, migration and contractile behavior. Finally, we demonstrate that the inhibition of TRPM4 activity alters cellular contractility *in vivo*, affecting cutaneous wound healing. Together, these findings provide the first evidence, to our knowledge, for a TRP channel specifically localized to focal adhesions, where it performs a central role in modulating cellular migration and contractility.

## Introduction

The actin cytoskeleton receives signals from the extracellular matrix (ECM) *via* cell-ECM adhesions referred to as focal adhesions (FAs). These adhesions influence cytoskeletal organization and actin polymerization, and these cytoskeletal structures in turn affect the formation and disassembly of FAs [1]. These reciprocal interactions coordinate adhesion signaling, mechanical stress, and the spatial dynamics of cytoskeletal organization, leading to changes in contractility properties and directional cell movement [1].

Over 160 gene products including protein kinases, protein phosphatases, proteases, scaffolding proteins and second messengers, collectively referred to as the “Adhesome”, have been identified as proteomic components of FAs [2–4]. These components transduce ECM-dependent signals to the actin cytoskeleton in response to mechanical stimulation, modulating the stability of FAs and actin cytoskeleton dynamics [4,5]. Integrin-based signaling at FAs activates several signaling pathways involving Ca^2+^ oscillations, protein kinases and small Rho GTPase family members, such as RhoA, Rac and Cdc42, and downstream effectors that coordinate FA dynamics and actin cytoskeleton reorganization, regulating the thickness of stress fibers, FA dynamics, and formation of lamellipodia and filopodia [4–6]. All of these processes regulate the traction forces on cell structure in response to changes in substrate, which lead to changes in FA size and actin cytoskeleton organization.

FA assembly and turnover play a key role in cell migration: early adhesions are associated with pathways that stimulate protrusion, whereas mature adhesions are associated with the development of tension [4]. The attachment provided by FAs also contributes to the proper traction forces required for cellular migration [4,5], which requires the coordinated formation of lamellipodia/protrusions at the leading edge, adhesion and detachment, cell contraction, and retraction at the trailing edge [7]. As such, FAs are critical structures that regulate the contractile properties of cells *via* regulation of the actin cytoskeleton. Moreover, regulation of FA turnover constitutes an important mechanism for tension transduction, cellular motility and contractility required for tissue remodeling during developmental and repair processes [1,4].

TRPM4 is a unique member of the TRP channel superfamily that conducts only monovalent cations, such as Na^+^ and K^+^. [8–10]. Interestingly, TRPM4 channel activity is required for cell migration [11–13], effects attributed to altered intracellular Ca^2+^ oscillations [11,12], and actin-based cytoskeleton dynamics are highly dependent on changes in Ca^2+^ levels [5]. However, the mechanisms and the downstream pathways involved in this process, and whether TRPM4 plays a more general role in cell migration remain unclear. Here, we show that TRPM4 localizes to FAs, and that TRPM4 channel activity contributes to FA turnover and lamellipodial actin cytoskeleton dynamics. Moreover, we provide evidence that TRPM4 activity regulates FAK and Rac GTPase activities, regulating cellular contractility and migration. Finally, we provide novel findings that link these effects of TRPM4 channel activity to the wound healing process. Together, these data suggest that the specific localization of TRPM4 at adhesion complexes underlies the spatial and temporal regulation of FA dynamics, cell contractility and migration.

## Materials and Methods

### Cell culture, plasmids, drug treatments and shRNA knockdown

Mouse Embryonic Fibroblasts (MEFs) were isolated from mouse embryos at stage E13 following the protocol described in [14]. The pregnant mice were used for mouse skin fibroblasts (MSFs) isolation as described in [15]. All animal use procedures were in strict accordance with the Chilean National Council for Sciences and Technology and were approved by the Institutional Animal Care and Use Committee of the Universidad de Chile (Protocol #0513 FMUCH). HEK293 and COS-7 were obtained from the American Type Cell Culture (ATCC) repository. TREx293-TRPM4 cells were generated as described [16]. MEFs, MSFs, HEK293, COS-7 and TREx293-TRPM4 cells were grown at 37 ˚C and 5% CO_2_ in DMEM High Glucose media (Invitrogen, Carlsbad, CA, USA) supplemented with 5% v/v fetal bovine serum (FBS). HUVEC cells [17] were cultured in DMEM High Glucose media supplemented with 20% v/v FBS. Plasmid encoding human TRPM4 FLAG-tagged (pcDNA4/TO-FLAG-hTRPM4) was a generous gift from Dr. Pierre Launay. Dr. Christopher Turner kindly gifted the EGFP-Paxillin plasmid. Plasmid encoding Rac1(Q61L) (pRK5-Rac1 L61) was acquired from Dr. Alan Hall (*via* Addgene, plasmid 15903). TRPM4 activity was modulated by treating the cells with 9-phenanthrol (Sigma-Aldrich, St Louis, MO, USA), a TRPM4 inhibitor [18]. TRPM4 knockdown was performed by transfecting the cells with shRNAs against murine TRPM4 (shRNA^TRPM4^) (Origene, Rockville, MD, USA). HEK293 cells were transiently transfected by using Lipofectamine 2000 (Invitrogen, Carlsbad, CA, USA) according the manufacturer’s instructions. MEFs cells were transfected by using Lipofectamine LTX reagent (Invitrogen, Carlsbad, CA, USA).

### Antibodies

For immunofluorescence and immunoblot experiments, we used as primary antibodies anti-TRPM4 (TA1008, Origene, Rockville, MD, USA) mouse monoclonal (mAb) (see S1 Fig for validation), anti-vinculin mouse mAb (V9131, Sigma, St Louis, MO, USA), anti-GFAP rabbit polyclonal (pAb) (G5601; Promega, Valencia, CA), anti-copine3 (TA308581, Origene), anti-cofilin rabbit mAb, anti-pY397 FAK rabbit mAb and anti-FAK rabbit mAb (5175, 8556 and 13009, respectively; Cell Signaling, Danvers, MA, USA) antibodies. For immunoprecipitation assays, we used anti-FLAG rabbit polyclonal antibody (F7425, Sigma, St Louis, MO, USA). For immunoblots we used anti-Grp75/mortalin mAb N52A/42 (UC Davis/NIH NeuroMab Facility, Davis, CA, USA) or otubulin (T5168, Sigma, St Louis, MO, USA) as a loading control. Alexa-conjugated secondary antibodies (Invitrogen, Carlsbad, CA, USA) were used for immunofluorescence labeling, and horseradish peroxidase-conjugated secondary antibodies (KPL, Gaithersbury, MD, USA) for immunoblotting.

### Immunoprecipitation assays

Immunopurification assays were performed according to [16]. Briefly, cells were plated at 50% confluency on 10 cm dishes and transfected with the pcDNA4/TO-FLAG-hTRPM4 plasmid. Forty-eight hours later, the cells were solubilized in lysis buffer containing 1% v/v Triton X-100, 150 mM NaCl, 10% v/v glycerol, 1 mM EDTA, 50 mM Tris-HCl (pH 7.4), 1 mM sodium orthovanadate, 5 mM NaF, 1 mM phenylmethylsulfonyl fluoride and protease inhibitor cocktail for 30 min at 4 ˚C, followed by centrifugation at 12,000 x g for 10 min at 4 ˚C. The supernatants were incubated with 5μg of anti-FLAG antibody overnight at 4 ˚C, followed by addition of protein A sepharose beads (GE Amersham, Piscataway, NJ, USA) for 1 h at 4 ˚C. The beads were washed five times in lysis buffer and used for subsequent assays, or immunopurified proteins eluted by boiling in reducing SDS sample buffer (RSB).

### Identification of TRPM4-associated proteins by LC-MS/MS analysis

The immunopurified complexes were resolved in 4%-12% gradient SDS-PAGE and stained with Colloidal Coomassie Blue staining. The bands were excised, diced and washed twice in 50% v/v acetonitrile /25 mM ammonium bicarbonate and dried 10 min in a speed vacuum concentrator. Cys residues were reduced by incubation of the gel pieces in 10 mM dithiothreitol (DTT) at 56°C for 1 h and protected by alkylation incubating with 55 mM iodoacetamide at room temperature for 45 min in the dark. Gel pieces were then washed 10 min in 50 mM ammonium bicarbonate, dehydrated in 50% v/v ACN/50 mM ammonium bicarbonate for 10 min and dried in a vacuum concentrator. Dried gel pieces were rehydrated in 50 mM ammonium bicarbonate on ice for 10 min, the solution was removed and the gel pieces were covered with 50 mM ammonium bicarbonate containing 10 ng/μL trypsin (Promega, Madison, WI, USA) and incubated overnight at 37°C. Digested peptide mixtures were extracted by vortexing with 50% v/v ACN/5% v/v formic acid for 30 min, and completely dried until further analysis. LC-MS/MS procedures were performed at the UC Davis Proteomics Facility using an ultra-performance liquid chromatography (UPLC) system (nanoACQUITY) coupled with an ion trap mass spectrometer (LTQ-FT, Finnigan) for LC-MS/MS data acquisition. MS/MS spectra were interpreted through the Mascot searches (Matrix Science) and Transproteomic Pipeline with a mass tolerance of 20 ppm, MS/MS tolerance of 0.4 or 0.6 D, and one missing cleavage site allowed.

### Immunofluorescence labeling

MEFs were incubated with DMSO (0.1% v/v final) vehicle, 10 μM 9-phenanthrol for 16 h. Cells were then fixed for 15 min at 4 ˚C in 4% w/v formaldehyde, 4% w/v sucrose in DPBS, and washed in DPBS. Cells were permeabilized and blocked with 0.1% v/v Triton X-100, 4% w/v nonfat dry milk in DPBS for 45 min at room temperature and labeled with anti-TRPM4 mAb and the F-actin-stain phalloidin (Cytoskeleton Inc., Denver, CO, USA). Primary antibodies were detected with Alexa-conjugated secondary antibodies (Invitrogen, Carlsbad, CA, USA). Images were acquired with a CCD camera installed on an Axiovert 200M microscope with 63X, 1.3 numerical aperture objective and an ApoTome coupled to Axiovision software (Zeiss, Oberkochen, Germany). Cells with lamellipodia were counted and analyzed as described [19].

### Focal adhesion isolation

FA complexes were isolated according the protocol described in [2]. Briefly, MEFs were plated at 50% confluency on 10 cm dishes coated with 10 μg/mL Collagen type I, and lysed in a hypotonic buffer (2.5 mM triethanolamine, pH 7.0) for 3 min. Cell bodies were removed by hydrodynamic force by using a Waterpik dental water jet (Interplak dental water jet WJ6RW, Conair). This cytosolic fraction (Cell Body fraction) was saved for further analyses. FAs attached to the plates were rinsed once with hypotonic buffer, collected by scraping and denatured in lysis buffer containing 1% w/v SDS (FA fraction), and sonicated for 30 s on ice. The proteins from both fractions were precipitated with 10% v/v trichloroacetic acid overnight at -80 ˚C. Precipitated proteins were resuspended in RSB 1X and resolved in SDS-PAGE. The efficiency of fractionation was determined by immunoblot by using anti-vinculin antibody as control.

### FA dynamics measurements and live cell imaging

FA turnover processes were studied by Live-Cell Time-Lapse recordings performed in MEFs transiently cotransfected with EGFP-Paxillin and shRNA^Scramble^ or shRNA^TRPM4^. Forty-eight hours post transfection, the cells were split in fibronectin-coated coverslips (10 μg/mL) and then serum-depleted for 16 h. FA assembly/turnover was induced by adding 5% v/v FBS in HBSS. Images were acquired every 1 min for 30 min. Images were processed using ImageJ software with the Time Series Analyzer v2.0 plugin. FA assembly and disassembly were visualized and quantified as the appearance or loss of fluorescence in a region of interest, according to [20]. The assembly and turnover rates were calculated by plotting the values of ln I/I_0_ *versus* time, according to method described in [21].

### Intracellular Ca^2+^ measurements

MEFs were seeded on 5 mm round coverslip coated with 2 μg/mL fibronectin (Sigma, St Louis, MO, USA) 24 h before the experiments. Cells were serum starved for 4 h before the experiments and then loaded with 2 μM Fura-2 at 37°C in HBSS. The coverslips were mounted in a recording chamber on an Olympus IX71 inverted microscope equipped with a Polychrome IV Imaging System coupled to a high-speed digital camera (Till Photonics, Pleasanton, CA). Images from Fura-2-loaded cells were acquired every 10 s by alternate excitation of light at 340 and 380 nm, and emission was captured at 510 nm using Imaging WorkBench 6.0 software (INDEC BioSystems, Santa Clara, CA). Chamber volume was maintained at ~400 μL. Cells were perfused with the HBSS at 37°C. The ratio of fluorescence at 340 nm to fluorescence at 380 nm was calculated, and all data are presented as the change in ratio units (∆F = ∆F_max_ + (∆F_max_—∆F_min_)).

### Immunoblot analysis and quantification

Immunoblot experiments were conducted by following the protocol described in [22]. The immunoblots were visualized by Pierce ECL Western Blotting Substrate (Thermo Scientific, Rockford, IL, USA) and quantified using NIH/ImageJ software.

### *In vitro* wound assays

*In vitro* wound assays were created in the confluent cell monolayer by manual scratching with a P200 pipette tip, washing once with DMEM 2% v/v FBS to remove loosely attached cells, and then cells were maintained in the same medium for 16 h. Cells were fixed and labeled with Phalloidin-Alexa 555 (Cytoskeleton Inc., Denver, CO, USA) and Hoechst 33258 (Invitrogen, Carlsbad, CA, USA). The width of the wound was measured and compared between the different conditions assayed.

### Boyden chamber transwell migration assays

Briefly, MEF-shRNA^Scramble^ and MEF-shRNA^TRPM4^ cells (1.0 x 10^4^ cells) were plated over Transwell chambers (8 μm pore, Corning Costar Corp, Tewksbury, MA, USA). Cell migration were induced by adding 10% v/v FBS in the lower chamber for 16 h at 37˚C. The non-migrating cells were removed and the migrating cells were fixed and stained with 0.2% w/v crystal violet/10% v/v ethanol. The migrating cells were counted and expressed as percentage of control.

### Three-dimensional culture and migration

In order to determine the role of TRPM4 in cellular invasion, 1.0x10^5^ of TREx293-TRPM4 was immersed in a collagen type I (1 mg/mL) in 48-wells plates as described in [23]. After incubation and polymerization of the collagen I matrix in DMEM/10% v/v FBS for 24 h, the contracted gels were immersed in a second collagen gel. Once the second gel polymerized, migration was induced by incubating the gels with 1% v/v FBS for 24 h. The migrating cells were counted and the total migration was expressed as cells/field and compared to the respective controls. In order to determine the role of TRPM4 in cellular contractility due changes in the actin cytoskeleton rearrangements, we used a 3D culture method [23]. Briefly, 1 mg/mL of soluble monomers of Collagen type I was mixed with 1.0x10^5^ cells of MEF-shRNA^Scramble^ and MEF-shRNA^TRPM4^ cells, neutralized with DMEM (4X) and polymerized at 37 ˚C for 1 h. Cellular contraction was induced with 5% v/v FBS. The contraction of the collagen matrix was evaluated after 48 h measuring the diameter of the gel and the data were expressed as the ratio of the area of the 48-well.

### Detection of activated Rac GTPase

For assaying Rac GTPase activity, G-LISA assay (Cytoskeleton Inc., Denver, CO, USA) was performed according to the manufacturer’s instructions.

### Skin explant culture

*Ex vivo* explant culture of 6 to 7 weeks old adult CJ1 mouse skin was performed as described previously [24]. The animals were anesthetized with Nembutal (50 mg/Kg) and the dorsal skin of the mice was shaved using a commercial shaver [25]. The skin was then washed with 70% v/v ethanol and the circular skin explants were removed by using 3-mm punches (Acuderm, Inc., Ft. Lauderdale, FL, USA) [24]. Explants were cultured for 2 d in 24-well dishes in DMEM/10% v/v over Collagen-coated 12 mm coverslips. At day 3 the explants were cultured in DMEM/5% v/v in presence of DMSO or 10 μM 9-phenanthrol for 8 d before fixing (4% w/v PFA for 15 min, 4% w/v sucrose in DPBS for 15 min at 4 ˚C).

### Zebrafish care, whole-mount in situ hybridization and wound healing assay

Embryos of zebrafish (*Danio rerio*) wild-type line Tübingen were obtained by natural spawning, raised at 28°C in embryo medium and staged according to morphology and age (hours and days post-fertilization; hpf and dpf) [26]. This line is available at the Fish Facility of the ICBM, Facultad de Medicina, Universidad de Chile. All animal use procedures were in strict accordance with the Chilean National Council for Sciences and Technology and were approved by the Institutional Animal Care and Use Committee of the Universidad de Chile (Protocol CBA# 0641 FMUCH). Whole-mount *in situ* hybridization was performed according to standard protocols [27] using antisense probes for *trpm4* genes based on the www.ensembl.org database (TRPM4b: ENSDART00000152447; TRPM4c: ENSDART00000092687). For wound closure assays, 72 hpf embryos were treated with 0.1% v/v DMSO or 20 μM 9-phenanthrol at 28°C in E3 medium for 4 h. The embryos (76 hpf) were then anesthetized (0.003% w/v tricaine), mounted in 1% w/v low melting agarose filled acrylic chambers and the tail was wounded with a 0.125 mm–diameter dissection needle (Fine Scientific Tools, Foster City, CA, USA). *In vivo* images of wound closure were recorded for 30 min every 2 min using Nikon Eclipse 80i microscopes mounted with Nikon Digital Sight DS-Fi1 digital camera connected to a computer running image capture software (NIS-Elements F3.0).

### Mouse model for cutaneous wound repair

The dorsal skin of pre anesthetized 6 to 7 weeks old CJ1 mice was shaved (n = 10). The skin was washed with 70% v/v ethanol and two wounds were made in the dorsal area using a punch biopsy of 3 mm (Acuderm, Inc.). A drop (~20 μL) of 20 μM 9-phenanthrol/20% w/v hydroxymethyl cellulose was topically administrated over the wound. As control, 0.1% DMSO/20% w/v hydroxymethyl cellulose was administrated. The wound area was measured at days 0, 1, 3, 5 and 7. All animal use procedures were in strict accordance with the Chilean National Council for Sciences and Technology and were approved by the Institutional Animal Care and Use Committee of the Universidad de Chile (Protocol #0513 FMUCH).

### Statistical analyses

All the experiments were repeated at least three independent times. The data were collected; quantified and expressed as the mean ± standard deviation. Normalized data were compared to respective control using Prism 4.0 (GraphPad Software, San Diego, CA, USA).

## Results

### TRPM4 interacts with proteins involved in actin cytoskeleton dynamics

**Fig 1:**
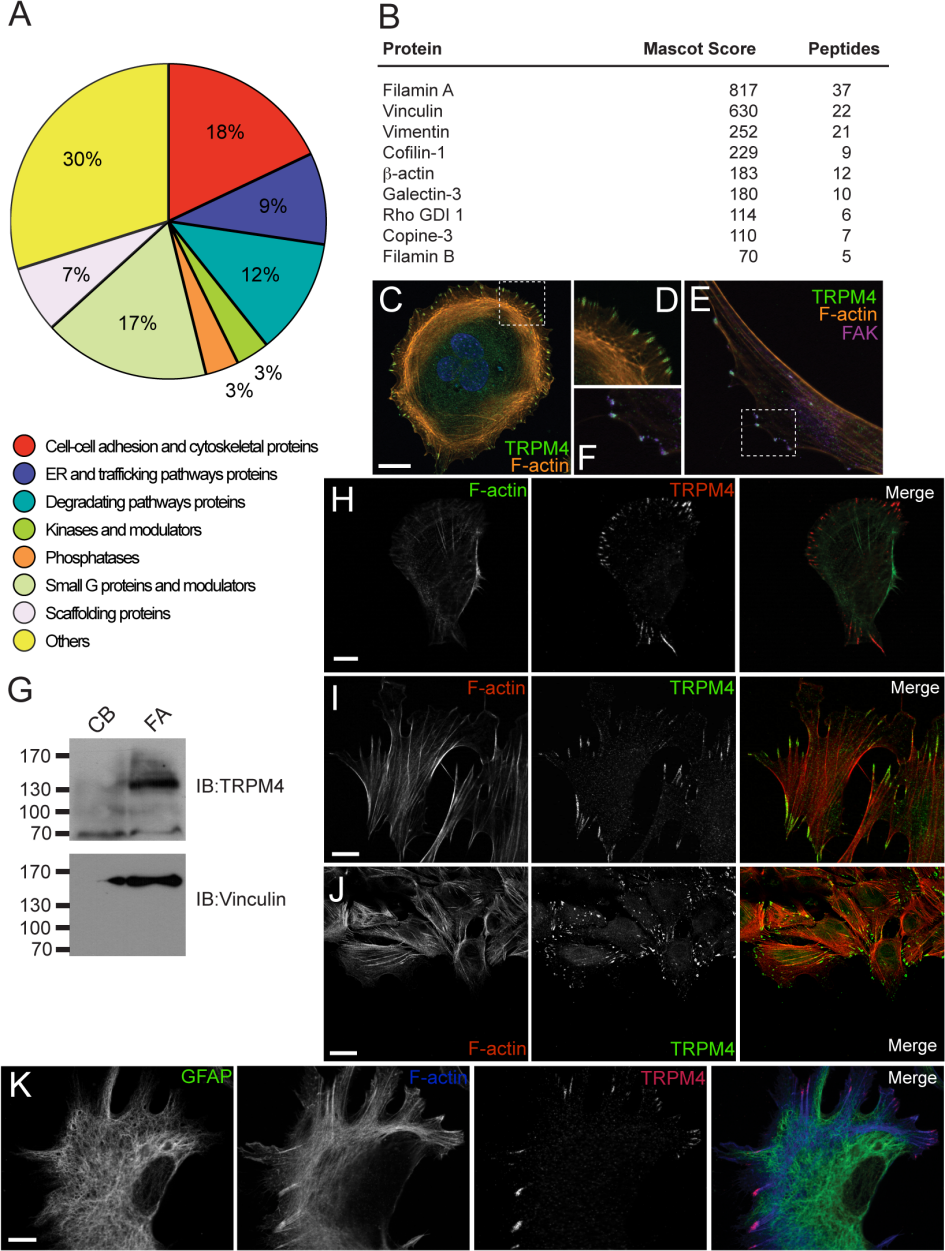
TRPM4 localizes to focal adhesions. A) Classification of putative TRPM4-associated proteins identified by LC-MS/MS. B) FA-related proteins identified as putative TRPM4 interacting proteins. C) TRPM4 localizes to FAs in MEFs. Cells were labeled with Hoechst (blue), mouse anti-TRPM4 mAb (green), and tRFP (red). D) Magnification of the section from C. E) TRPM4 (green) colocalizes with Focal Adhesion Kinase (FAK, magenta) in MEFs. F) Amplification of the region marked in E. G) Biochemical isolation of FA complexes (FA) from MEFs. The cell body fraction is labeled as CB. TRPM4 localizes to FAs in mouse pancreatic (H) and skin fibroblasts (I); Human Umbilical Vein Endothelial Cells (HUVEC) (J) and astrocytes (K). Scale bar: 5 μm.

To identify novel TRPM4-associated proteins, we immunopurified FLAG-tagged human TRPM4 protein-containing complexes from HEK293 cells and analyzed their components by LC-MS/MS. We identified 124 proteins with a Mascot Score >50 as candidate interacting partners. Further classification of these proteins based on their known cellular function indicated that the largest group of proteins (18% total) have cytoskeleton-associated functions (Fig 1A). Interestingly, nine of these proteins are related with the establishment of FAs and cellular attachment (Fig 1B).

Given these results, we next addressed whether TRPM4 localizes at FA complexes. We determined the cellular distribution of endogenous TRPM4 protein in mouse embryonic fibroblasts (MEFs), and found that TRPM4 is localized in a punctuate pattern at the cell periphery, coincident with the tips of stress fibers, and resembling the localization of FAs (Fig 1C and 1D). We found that TRPM4 colocalizes with the FA marker focal adhesion kinase (FAK) at the tips of the stress fibers (Fig 1E and 1F). Similar to the FA component vinculin, TRPM4 was also enriched in FAs mechanically isolated from MEFs relative to the cytoplasmic/cell body fraction or CB (Fig 1G). We found a similar FA-associated localization of TRPM4 in a variety of primary cell cultures, including mouse pancreatic fibroblasts (Fig 1H), mouse skin fibroblasts (MSFs) (Fig 1I), human umbilical vein endothelial cells (HUVEC) (Fig 1J) and rat astrocytes (Fig 1K).

### TRPM4 regulates FA disassembly

**Fig 2:**
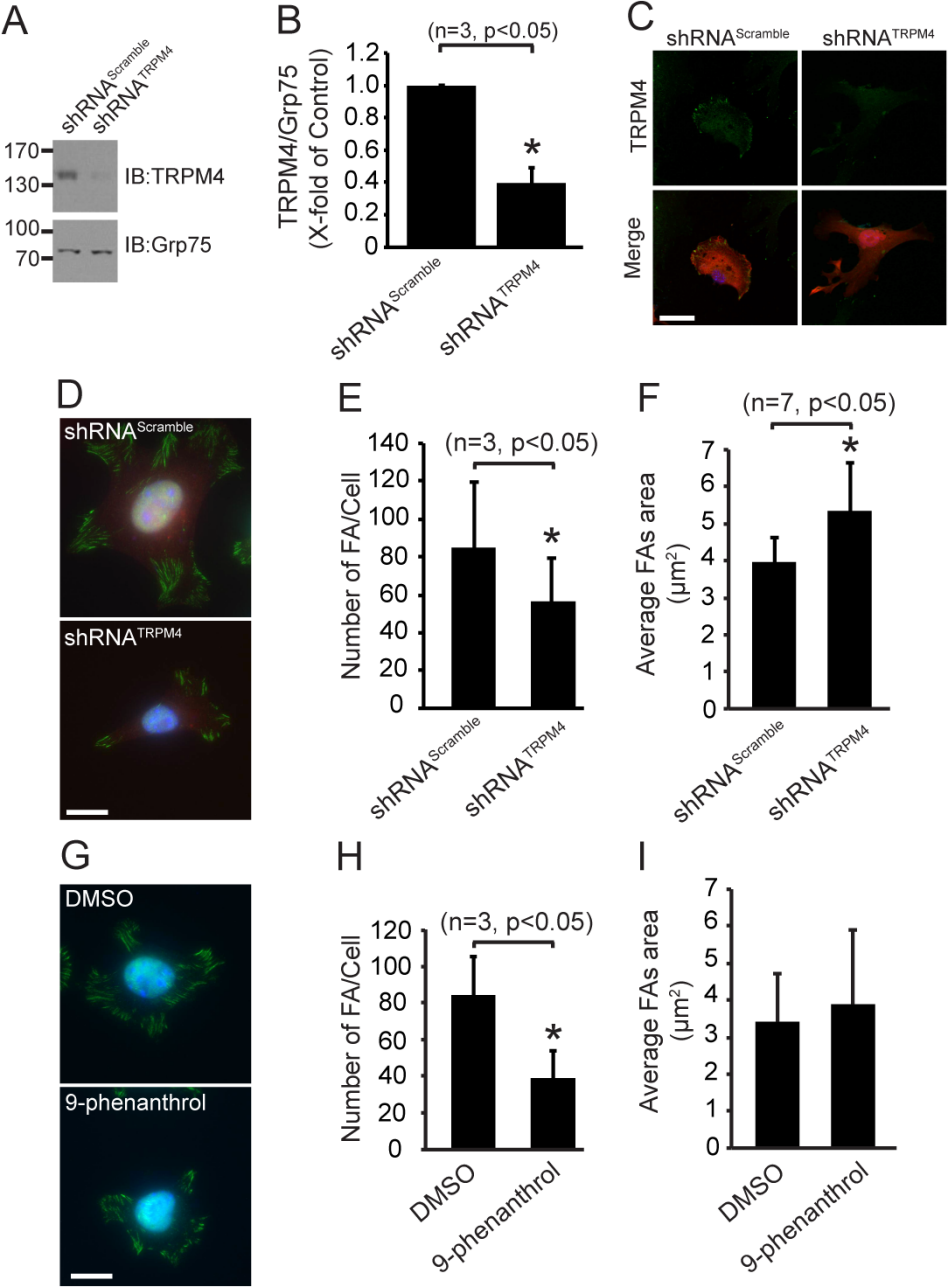
TRPM4 regulates the number and size of focal adhesions. A) Representative immunoblot from MEFs subjected to shRNA-mediated TRPM4 knock down. Membranes were incubated with mouse anti-TRPM4 mAb, and anti-Grp75 mAb as a loading control. B) Graph of the densitometric analyses of three independent immunoblot experiments. Statistical analysis was performed using a Mann Whitney test. C) Immunofluorescence labeling of MEFs transfected with shRNA^Scramble^, and shRNA^TRPM4^. Cells were labeled with Hoechst (blue), and mouse anti-TRPM4 mAb (green); tRFP (red) was used as transfection marker. Scale bar corresponds to 10 μm. D) Immunofluorescence labeling of MEFs transfected with shRNA^Scramble^ and shRNA^TRPM4^. Cells were labeled with Hoechst (blue), and mouse anti-vinculin mAb (green); tRFP (red) was used as transfection marker. Scale bar corresponds to 10 μm. Quantification of FA number (E) and areas (F) from the shRNA-transfected cells (n = 15 cells for shRNA^Scramble^ and n = 15 cells for shRNA^TRPM4^ from 7 independent experiments). G) Immunofluorescence labeling of MEFs treated with DMSO (0.1% v/v) and 10 μM 9-phenanthrol. Cells were labeled with Hoechst (blue) and mouse anti-vinculin mAb (green). Scale bar corresponds to 10 μm. Graphs of FA number (H) and areas (I) are shown (20 cells per condition, n = 3, p<0.05). The number and areas of the FAs were analyzed using NIH/ImageJ software. The graphs correspond to mean ± standard deviation. Statistical analysis was performed using a Mann-Whitney test.

To define the role of TRPM4 in FA function, we performed shRNA-based TRPM4 knockdown (Fig 2A and 2C) and measured the number of FAs. We found that TRPM4 knockdown caused a diminished number of FAs (Fig 2D and 2E). Moreover, we found that TRPM4 silencing caused a ~34% increase in FA size (Fig 2F). Interestingly, pharmacological inhibition of TRPM4 with 9-phenanthrol, a potent inhibitor of TRPM4, caused similar effects on the number of FAs (Fig 2G and 2H), as well as a ~13% increase in the average FA size (Fig 2I).

**Fig 3:**
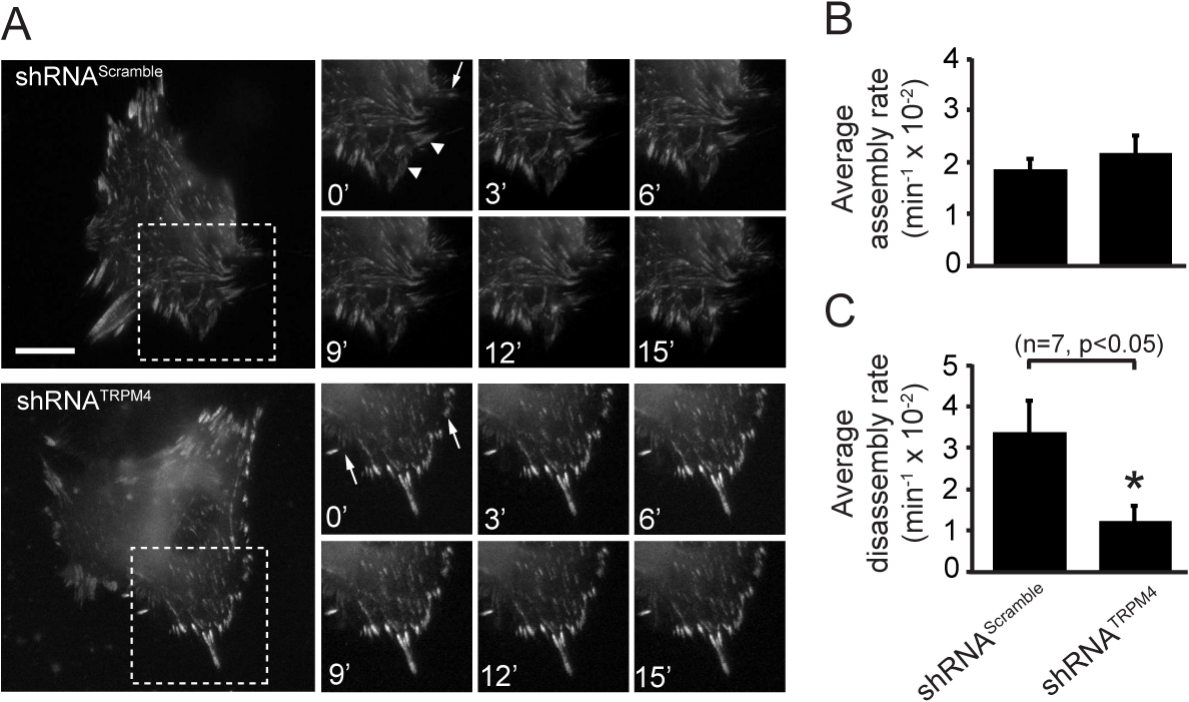
TRPM4 regulates FA turnover. (A) Representative time-lapse imaging from MEFs coexpressing shRNA^Scramble^ or shRNA^TRPM4^ with EGFP-Paxillin. Assembling FAs are marked with arrows. Arrowheads mark FAs undergoing turnover. Scale bar: 5 μm. Quantification of assembly (B) and disassembly rates (C) of FAs from live-cell time-lapse recordings of EGFP-Paxillin transfected MEFs. Statistical analysis was performed using a Mann-Whitney test from 7 independent experiments.

The increased size of FAs upon TRPM4 knockdown might be caused by reduced FA disassembly rates. We next assessed the effects of TRPM4 knockdown on FA dynamics in time-lapse experiments (Fig 3A–3C and S1 and S2 Movies), from which we measured FA assembly and disassembly rates. Interestingly, we did not observe significant differences in assembly rates in the FAs that did assemble in the TRPM4-depleted cells (shRNA^Scramble^: 0.0158 ± 0.0019 min^-1^ versus shRNA^TRPM4^: 0.0217 ± 0.0033 min^-1^) (Fig 3A). However, we observed significantly diminished disassembly rates of FA complexes in shRNA^TRPM4^–transfected cells (shRNA^Scramble^: 0.0299 ± 0.0092 min^-1^ versus shRNA^TRPM4^: 0.0123 ± 0.0035 min^-1^, p< 0.05) (Fig 3B). These results suggest that TRPM4 activity regulates the rate of FA disassembly but not assembly.

### TRPM4 regulates intracellular Ca^2+^ signaling and activation of FAK

**Fig 4:**
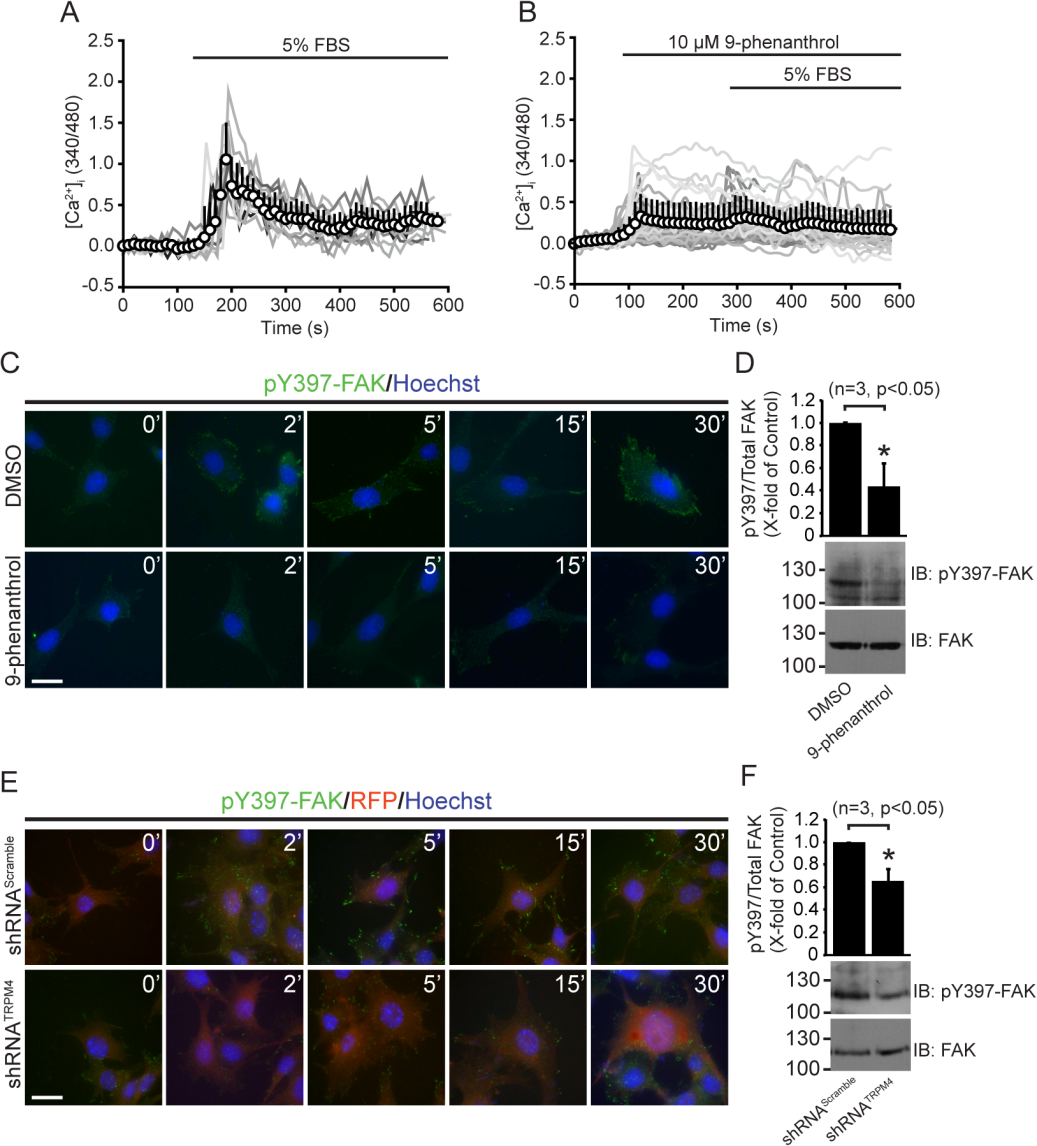
TRPM4 regulates intracellular Ca^2+^ signaling and activation of FAK. Time courses for normalized fluorescence in MEFs loaded with Fura-2 and treated with DMSO (0.1%v/v) (A) and 10 μM 9-phenanthrol (B). Gray lines correspond to [Ca^2+^]_i_ changes in individual MEFs (12 cells, 6 independent experiments for Control; 48 cells, 6 independent experiments for 9-phenanthrol treatment). White circles correspond to the mean trace of [Ca^2+^] signals. TRPM4 activity is required for FAK activation. (C) Time-course activation of FAK upon TRPM4 pharmacological inhibition. Cells were depleted for 4 h and stimulated with 10% v/v serum in presence of DMSO and 10 μM 9-phenanthrol. Cells were fixed at 0, 2, 5, 15, 30 and 60 min post-treatment. Then, the cells were stained with Hoechst (blue) and rabbit mAb anti-pY397-FAK (green). Representative images from three independent experiments of MEFs treated with DMSO and 10 μM 9-phenanthrol are shown. Scale bar correspond to 5 μm. (D) Representative immunoblot from MEFs treated with DMSO and 10 μM 9-phenanthrol is shown. Cells were depleted for 4 h and then, stimulated with 10%v/v serum for 30 min. Upper graph corresponds to the densitometric analyses of 3 independent experiments. Statistical analysis was performed using a Mann Whitney test. (E) Time-course activation of FAK upon TRPM4 silencing. MEF-shRNA^Scramble^ and MEF-shRNA^TRPM4^ were treated as Fig 4C, and stained with Hoechst (blue) and rabbit mAb anti-pY397-FAK (green). tRFP (red) was used as transfection marker. Representative images from three independent experiments are shown. Scale bar correspond to 5 μm. (F) Representative immunoblot from MEFs transfected with shRNA^Scramble^ and shRNA^TRPM4^ is shown. Cells were treated as Fig 4D. Upper graph corresponds to the densitometric analyses of 3 independent experiments. Statistical analysis was performed using a Mann Whitney test.

Calcium is a key signal and effector for FAs dynamics [28], and as TRPM4 channels modulate Ca^2+^ signals and oscillations in a number of different cell types [29], TRPM4 could affect FA by influencing intracellular Ca^2+^ dynamics. To test this possibility, we stimulated MEFs with serum, which induces FA turnover, and measured intracellular Ca^2+^ concentration ([Ca^2+^]_i_). Serum stimulation induced a rapid and transient [Ca^2+^]_i_ increase (Fig 4A). Interestingly, preincubation with 10 μM 9-phenanthrol abolished the serum-induced [Ca^2+^]_i_ increase (Fig 4B), suggesting a crucial role for TRPM4 in regulating the initial Ca^2+^ influx leading to FA turnover.

As these early Ca^2+^ increases contribute to activation of FAK [30], which plays a fundamental role in the subsequent events of FA turnover, cell migration and contractility [31], we next determined whether TRPM4 regulates FAK activity. Serum-starved MEFs were stimulated with serum in the presence of 10 μM 9-phenanthrol. We analyzed autophosphorylation of the Tyr397 residue of FAK (pY397-FAK) that is required for FAK activation, and found that TRPM4 inhibition diminished serum-stimulated phosphorylation of Tyr397-FAK (Fig 4C and 4D). Similar results were observed in shRNA-transfected fibroblasts (Fig 4E and 4F).

### TRPM4 channels regulate cellular spreading by regulating Rac GTPase activation

**Fig 5:**
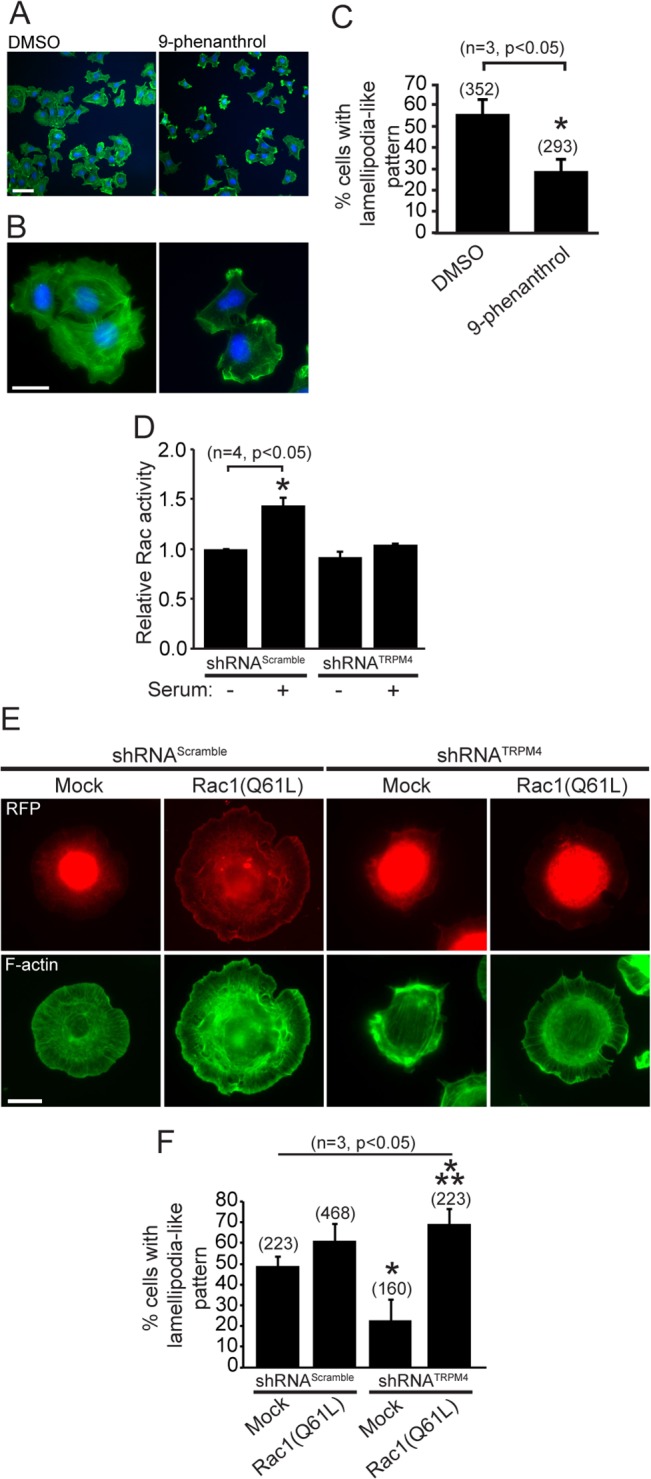
TRPM4 channels regulate Rac GTPase activity. A) Cells were incubated with DMSO and 10 μM 9-phenanthrol for 40 min on Collagen-coated coverslips. Cells were fixed and labeled with the F-actin stain Alexa 488 phalloidin (green) and Hoechst (blue). (Scale bar: 10 μm). B) Magnification of cells in A. (Scale bar: 5 μm). C) Graph shows the percentage of cells with lamellipodia-like pattern. Data are from 8 independent coverslips from 3 independent experiments. *, significant difference (p<0.05) versus DMSO controls. Statistical analysis was performed using a Mann-Whitney test. D) Rac GTPase activity of MEF-shRNA^Scramble^ and MEF-shRNA^TRPM4^ determined using a G-ELISA commercial kit. Rac GTPase activity was stimulated with 5% v/v serum for 30 minutes. *, significant difference (p<0.05) versus shRNA^Scramble^ controls. Statistical analysis was performed using a two-way ANOVA test. E) Expression of Rac1(Q61L) rescues the loss of lamellipodial formation caused by the depletion of TRPM4. F-actin was labeled with the F-actin stain Alexa 488 phalloidin (green). RFP (red) corresponds to shRNA-transfected cells. (Scale bar: 5 μm). F) Quantification of the percentages of cells with lamellipodia-like phenotype from experiments showed in E. The graph corresponds to data collected from 3 independent experiments (2 coverslips per condition). *, significant difference (p<0.05) versus shRNA^Scramble^/Mock controls. **, significant difference (p<0.05) versus shRNA^TRPM4^/Mock. Statistical analysis was performed using a two-way ANOVA test.

FA formation and turnover constitute key processes for proper interaction of cells with ECM [4]. Moreover, early Ca^2+^ increases and FAK activation are required for the actin cytoskeleton reorganization involved in cellular attachment and lamellipodia formation processes [5]. Therefore, we sought to determine the role of TRPM4 in cellular adhesion. We observed a diminished cell size upon pharmacological inhibition of TRPM4, when compared to control cells (Fig 5A and 5B). Moreover, we found changes in the distribution of the actin cytoskeleton after the cell adhesion process, with a diminished lamellipodia-like actin distribution in the 9-phenanthrol-treated cells (Fig 5C), suggesting that TRPM4 contributes to proper cell spreading during the cell adhesion process, and to actin cytoskeleton reorganization.

The small GTPase Rho family member Rac is involved in generating actin-rich extensions known as lamellipodia and ruffles, a process initiated by peripheral actin polymerization [32]. Lamellipodia formation is required for both cell spreading and migration, and FAK regulates Rac GTPase activity during these processes [33,34]. We next assessed to determine whether Rac GTPase is a downstream molecular target of TRPM4 activity in serum stimulated shRNA-transfected MEFs. While we did not observe significant differences in Rac activity between shRNA^Scramble^ and shRNA^TRPM4^-transfected cells in the absence of serum stimulation (Fig 5D), we found that TRPM4 knockdown caused a robust inhibiton (~73%) of the serum-induced increase in Rac activity. In addition, we observed diminished lamellipodia-like actin distribution in the shRNA-transfected fibroblasts (Fig 5E and 5F). Moreover, we found that cotransfection with a constitutively active Rac1 (Rac1 Q61L) rescued the deficiency in lamellipodia seen in the TRPM4 knockdown cells (Fig 5E and 5F). These data suggest that TRPM4 activity regulates cellular spreading through effects on Rac GTPase activity.

### TRPM4 regulates cellular migration

**Fig 6:**
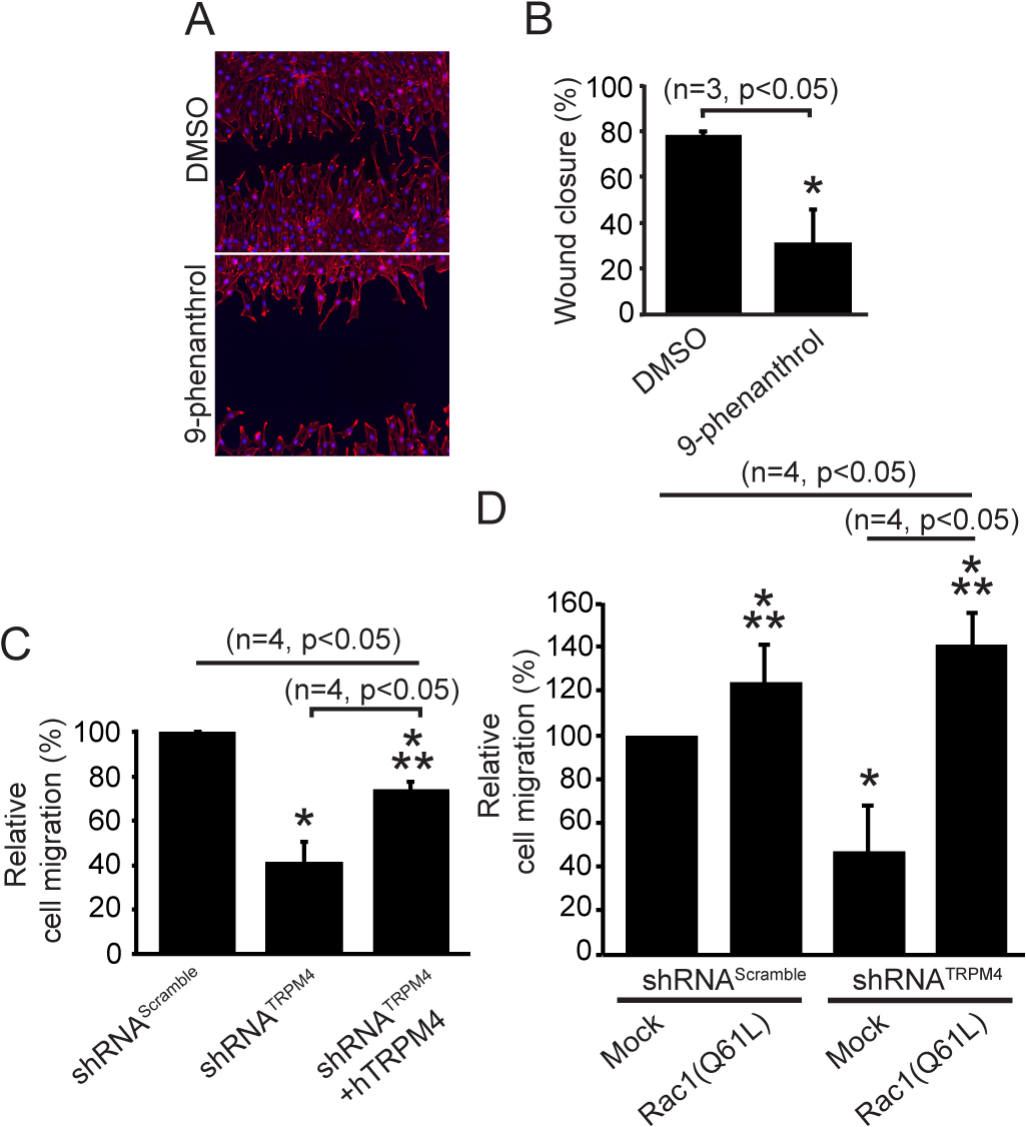
TRPM4 controls cellular migration *via* Rac1 GTPase activity. A) MEFs were incubated with DMSO (0.1% v/v final) vehicle or 10 μM 9-phenanthrol for 16 h. F-actin was labeled with the F-actin stain Alexa 555 phalloidin (red) and Hoechst (blue). B) Quantified results from A. The percentage of wound closure is expressed as mean ± standard deviation; s.d. (n = 3, p<0.05). *, significant difference (p<0.05) versus DMSO controls. Statistical analysis was performed using a Mann-Whitney test. C) Graph from Transwell Boyden chamber migration assays of MEFs transfected with shRNA^Scramble^ and shRNA^TRPM4^. Cells were stimulated with 10% v/v serum for 16 h. The bars represent the mean ± s.d. (n = 3; p<0.05 compared to control). *, significant difference (p<0.05) versus shRNA^Scramble^ controls. Statistical analysis was performed using a two-way ANOVA test. D) Graph from Transwell Boyden chamber migration assays of MEFs transfected with shRNA^Scramble^ and shRNA^TRPM4^ and coexpressing Rac1(Q61L). Cells were stimulated with 10% v/v serum for 16 h. The bars represent the mean ± s.d. (n = 3; p<0.05 compared to control). *, significant difference (p<0.05) versus shRNA^Scramble^/Mock controls. **, significant difference (p<0.05) versus shRNA^TRPM4^/Mock controls. Statistical analysis was performed using a two-way ANOVA test.

We next sought to determine whether TRPM4 could affect migratory behavior in fibroblast cells. We first studied the effects of TRPM4 inhibition on the migratory phenotype by performing wound migration assays in MEFs treated with 9-phenanthrol. We observed a diminished migratory behavior in the 9-phenanthrol-treated cells compared to DMSO-treated (Fig 6A and 6B).

We next performed Transwell Boyden chamber migration assays on MEFs transfected with shRNA–encoding plasmids targeting TRPM4. We observed a ~50% decrease on the migratory phenotype in cells expressing shRNA^TRPM4^ transfected cells compared to control shRNA^Scramble^-transfected cells (Fig 6C). Importantly, the cotransfection of the shRNA-resistant FLAG-hTRPM4 together with shRNA^TRPM4^ substantially rescued the migratory phenotype (Fig 6C), demonstrating a crucial role for TRPM4 in cell migration. Moreover, a constitutively active Rac1 mutant (Rac1 Q61L) rescued the lack of migratory behavior in shRNA^TRPM4^-transfected cells (Fig 6D), demonstrating that Rac1 activity is downstream of the effects of TRPM4 on cellular migration.

### TRPM4 regulates cell invasion and remodeling in 3D matrices

**Fig 7:**
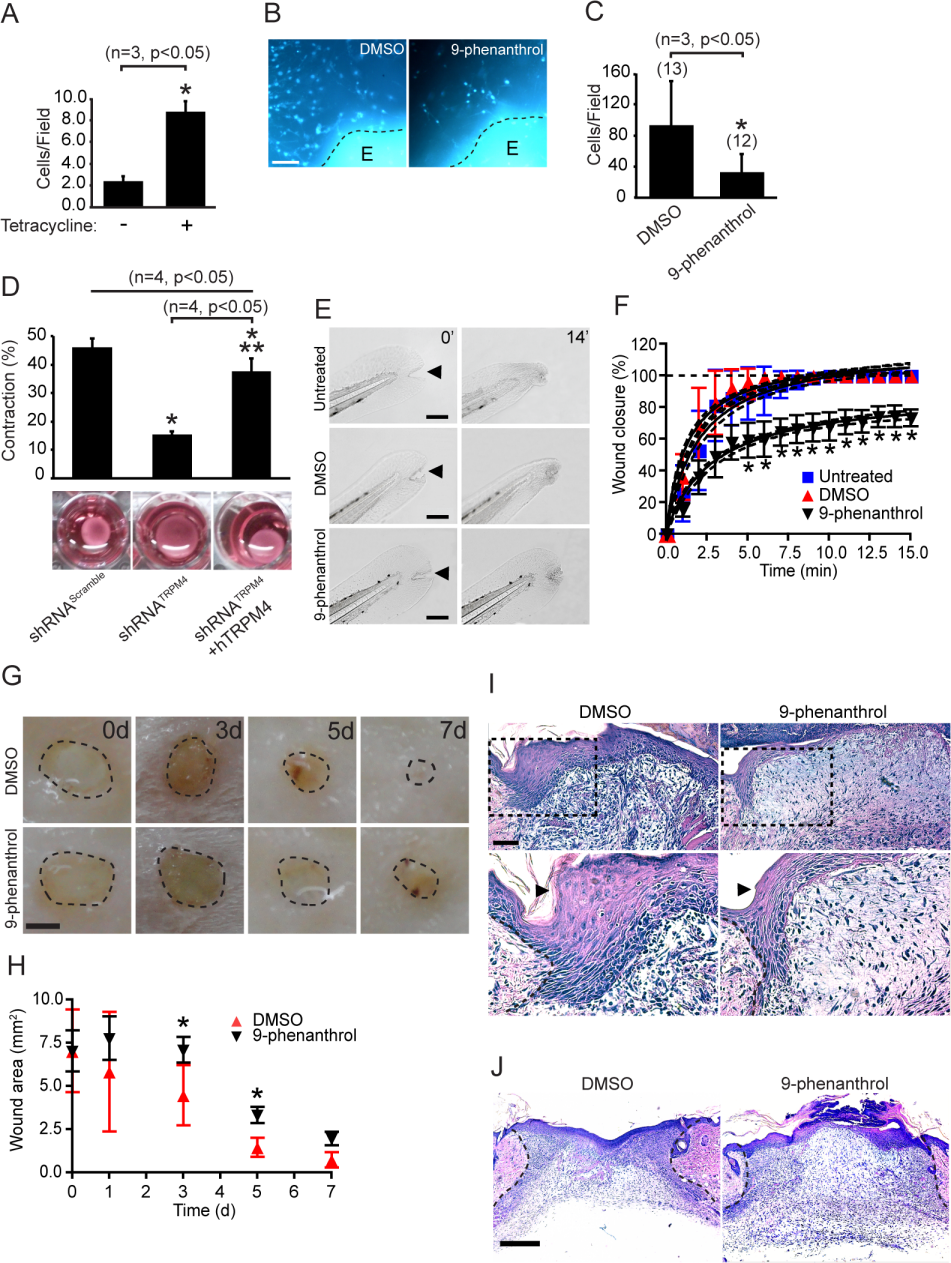
TRPM4 promotes cellular contractility and wound healing. A) Three-dimensional (3D) invasion assay of TREx293-TRPM4 cells. TRPM4 expression was induced by adding 1 μg/mL Tetracycline to the media (n = 3, p<0.05 compared to control). *, significant difference (p<0.05) versus non-stimulated cell control. Statistical analysis was performed using a Mann-Whitney test. B) Fibroblasts migration from skin grafts (E) treated with DMSO or 20 μM 9-phenanthrol. Grafts were fixed 5 days after explant and labeled with Hoechst (blue). Scale bar: 1 mm. C) Quantification of the experiment shown in (B). The data correspond to cell counts from 3 independent experiments (13 and 12 explants for DMSO and 9-phenanthrol treatments, respectively). *, significant difference (p<0.05) versus DMSO controls. Statistical analysis was performed using a Mann-Whitney test. D) Three dimensional contraction assay of MEFs transfected with shRNA^Scramble^ and shRNA^TRPM4^. Contraction was induced by incubating the immersed cells with 10% v/v serum for 48 h. The upper graph represents the collected data for 4 independent assays. *, significant difference (p<0.05) versus shRNA^Scramble^ controls. **, significant difference (p<0.05) versus shRNA^TRPM4^. Statistical analysis was performed using a two-way ANOVA test. E) Frames (t = 0 and 14 min) from time lapse of untreated, DMSO and 9-phenanthrol treated wounds in zebrafish tails. Scale bar: 1 mm. F) Quantification of the wound closure experiments from (E) (n = 10 larvae per condition). Statistical analysis was performed using a two-way ANOVA test. G) Excisional cutaneous wounds were created using a 3 mm biopsy punch. Images from the time course of wound closure in the presence of DMSO (control) and 9-phenanthrol (n = 5 mice). Scale bar: 1.5 mm. H) Wound closure was monitored measuring the area of the wound on the indicated days post-wounding. Statistical analysis was performed using a two-way ANOVA test. I) Images of skin wounds at 3 days post-wounding. Bottom panels show magnifications of the areas marked in the upper panels. Arrowheads mark the epithelial tissue. Scale bar: 50 μm. J) Images of wounds at 5 days post-wounding. The dashed lines indicate the limit of the wound area. Scale bar: 500 μm.

We next assessed the role of TRPM4 on cell invasion in a realistic 3D cell culture model using low invasive HEK293-derived (TREx293-TRPM4) cells into collagen I matrices. Induction of TRPM4 expression resulted in a 4-fold increase in cell invasion, compared to the non-induced control cells (Fig 7A). Therefore, we conclude that TRPM4 contributes to the cellular invasion. Moreover, we observed that in mouse skin grafts immersed in a collagen I matrix, the control explants (*i*.*e*. DMSO) presented an extended outgrowth (Fig 7B and 7C), with a clear expression of TRPM4 in the FAs of the migrating cells (S2 Fig). Conversely, 9-phenanthrol-treated explants developed a diminished outgrowth, consistent with the role of TRPM4 in cell invasion (Fig 7B and 7C). We also observed a diminished contractile phenotype in shRNA^TRPM4^-transfected cells compared to the shRNA^Scramble^-transfected cells (Fig 7D), and cotransfection of FLAG-hTRPM4 with the shRNA^TRPM4^ rescued cell contractility (Fig 7D). Together, these results suggest that TRPM4 regulates the cellular migration and contractility.

### Pharmacological inhibition of TRPM4 delays skin wound healing

Regulation of cell migration and contractility mediated by FAK and Rac activities are also key processes in wound repair [35,36]. Since TRPM4 is widely expressed in skin of zebrafish (S3 Fig and [37]), we used this model to determine whether pharmacological inhibition of TRPM4 affected wound closure. Interestingly, larvae incubated with 9-phenanthrol displayed a reversible phenotype with reduction of cardiac rhythm and escape response (data not shown), consistent with previous described effects for TRPM4 inhibition [18,38]. We also observed a ~30% reduction in wound closure in 9-phenanthrol-treated larvae compared to the control group (9-phenanthrol: 70.53 ± 8.57% of wound closure, p<0.001; Fig 7E and 7F, and S3–S5 Movies). In addition, we found that the initial rate of closure is diminished in the 9-phenanthrol-treated larvae (Untreated: 26.80 ± 1.54% closure min^-1^, DMSO: 30.27 ± 1.48% closure min^-1^ and 9-phenanthrol: 16.69 ± 0.71% closure min^-1^, p<0.001; Fig 7F). Moreover, we observed a delay in reaching the maximal closure in the 9-phenanthrol-treated larvae (Untreated: 5.80 ± 2.84 min, DMSO: 5.05 ± 1.53 min and 9-phenanthrol: 13.00 ± 2.91 min, p<0.001). These data suggest a role for TRPM4 activity during wound healing.

We next used a cutaneous wound healing mouse model to investigate the possible role of TRPM4 in wound closure (Fig 7G). We found significant decreases in the wound closure of the 9-phenanthrol-treated tissues at 3 and 5 days post-injury (Fig 7G and 7H). Further histological examination of the day 3 and 5 wounds revealed significant delay in the wound closure of the 9-phenanthrol-treated tissues (Fig 7I and 7J). These wounds showed defective re-epithelialization and formation of granulation tissue (Fig 7I and 7J), consistent with a diminished migration of these cellular lineages. On day 3, control wounds present complete re-epithelialization, presence of inflammatory infiltrate, ingrowth fibroblast and loose connective tissue deposition (Fig 7I). Conversely, incomplete re-epithelialization, inflammatory infiltrate and presence of fibrin were observed in the wounds treated with 9-phenanthrol (Fig 7I). We also observed a diminished contraction in the 9-phenanthrol-treated wounds at day 5, a process caused by the retraction of the granulation tissue (Fig 7J), consistent with our results observed in the 3D matrices (Fig 7D). These findings suggest a role of TRPM4 in the wound closure.

## Discussion

Cellular migration requires specific localization of the different components of its varied signaling effectors, such as protein kinases, phosphatases and ion channels. Despite the fact that several ion channels have been described as regulators of focal adhesion/cytoskeleton dynamics and mechanotransduction, only a few of these proteins have been demonstrated as intrinsic components of the adhesome [3]. In this study, we demonstrate that TRPM4, a unique member of the TRP channel family that conducts only monovalent cations [8], is the first member of this superfamily that localizes at focal adhesions, where it contributes to the regulation of the number and size of these complexes by modulating their disassembly dynamics. Moreover, we demonstrate that TRPM4 regulates FAK and Rac activation.

A role for TRPM4 in cellular migration has been previously established. Barbet *et al*. (2008) demonstrated that TRPM4 regulates dendritic cells migration [11]. In addition, Shimitzu *et al*. (2008) reported that TRPM4 modulates the migration of mast cells [12]. These effects are mediated by a TRPM4-dependent regulation on the Ca^2+^ oscillations. Consistently, we found that TRPM4 regulates the migration of fibroblasts and serum-induced [Ca^2+^]_i_ increases. Spatiotemporal regulation in [Ca^2+^]_i_ dynamics is an important process in the signaling associated to cell migration and contractility [28] and plays a major role in actin cytoskeleton [4,5,28]. Interestingly, the activity of several proteins involved in FAs and cytoskeletal dynamics is regulated by changes in [Ca^2+^]_i_ [28]. Several Ca^2+^ channels involved in these processes have been identified [28], however, a detailed description of their regulatory mechanisms has not been accomplished. Although TRPM4 itself is Ca^2+^ impermeable, this ion channel regulates increases in [Ca^2+^]_i_ in several cell types [8,11,29,39]. Recently, Gonzales *et al*. (2014) suggested that in smooth muscle cells, mechanical stimulation causes the activation of TRPC6, triggering activation of TRPM4 and leading to increased [Ca^2+^]_i_ *via* activation of voltage-dependent Ca^2+^ channels (VDCCs) [40]. Interestingly, VDCCs regulate migration and contractility in fibroblasts [41]. In our experiments, we observed that serum stimulation causes a fast and transient increase in [Ca^2+^]_i_, which is consistent with a possible role of VDCC activation. Moreover, our results suggest that 9-phenanthrol treatments abolished the serum-induced [Ca^2+^]_i_ increase, decreased FA number and increased in their size. Interestingly, similar effects on FAs have been found in response to inhibition of Ca^2+^ signaling [42]. Thus, our data suggest that localized Na^+^ influx at FAs through TRPM4 would cause fast membrane depolarization, and that in turn could activate VDCCs. We propose that in this manner, TRPM4 localized at FAs mediates coordinated changes in [Ca^2+^]_i_, regulating FA turnover, and impacting cell migration and contractility. Colocalization with other ion channels that have been reported as intrinsic focal adhesions components, such as K^+^ channels like Kir4.2 [43] and Kv11.1 [44], might then provide a fine regulatory mechanism for the TRPM4-dependent depolarization.

Our data demonstrate that TRPM4 regulates FA disassembly rate. Our results suggest that the effects of TRPM4 on cellular migration are related to its role in the regulation of the FA turnover. Here, we demonstrate that TRPM4 regulates FAK and Rac. FAK and Rac activities are key regulators of FA turnover, cell migration and contractility [45]. Increases in [Ca^2+^]_i_ induce the turnover of FAs *via* regulation of paxillin and FAK activities, and acting through Rac activity [28,34,46,47]. FAK activity is required for FA turnover *via* ERK/MLCK activation, affecting cellular migration and contractility [21]. Moreover, effects similar to our findings have been described for manipulating Rac activity [48]. Several reports have demonstrated that the specific activation of Rac through different GTP exchange factors (GEF), such as Dock180, Asef2 and STEF, plays a critical role in FAs number, size and turnover [48–50]. Moreover, a recent role for Rac activity in cellular contractility has been elucidated [50]. Our results suggest that TRPM4-triggered Ca^2+^ increases could activate FAK and Rac, which regulate cellular migration and contractility processes. Future studies are required to identify the GEF involved in the activation of Rac that is dependent on TRPM4.

TRP channels have emerged as novel regulators of cellular migration [51]. Other members of this family, such as TRPM7, have been shown to be involved in FA regulation [28]. However, no direct evidences for their localization at these complexes have been provided. Our data demonstrate the specific localization of TRPM4 in FAs. These results support the notion that the specific localization of ion channels at discrete sites exerts key functions in fundamental physiological processes, as important constituents of signaling platforms required for proper cellular function, and as local mediators of these events. The structural basis of TRPM4 localization in FAs constitutes an intriguing question. Despite the large amino- and carboxyl- terminal regions (~680 and 200 residues, respectively), no clear protein-protein interaction domains can be identified within its primary sequence. Further analysis of TRPM4 structural features will help to answer these questions.

In addition to their ion conducting functions, ion channels serve as platforms for organizing macromolecular complexes that can participate in different transduction pathways [52,53]. Therefore, ion channels may not only participate in cellular homeostasis as “pores”, but they also could modulate cell-signaling pathways *via* the extensive cytoplasmic domains that are a distinct feature of their structure [54–56]. Indeed, other TRP members have scaffolding functions. For instance, in the *Drosophila* eye, TRP plays a crucial role as a scaffolding protein regulating the localization of INAD [54,57]. Moreover, TRPV1 modulates filopodia formation independent of its conducting properties. In this study, we identified 124 putative TRPM4-interacting proteins through a mass spectrometry-based approach. These included FA proteins, cytoskeletal proteins, and protein kinases and phosphatases. We attempted to validate some of the putative interactions, which could have potential regulatory interest. Therefore, we assayed the TRPM4 coimmunoprecipitation with vinculin, a key component of the FAs, cofilin 1, a protein involved in the polymerization of actin fibers [58], and copine 3, a novel Ca^2+^-binding protein, which regulate focal adhesions stability [59]. We did not detected coimmunoprecipitation between TRPM4 and those proteins (S3 Fig). We hypothesize three possible reasons for these results: 1) TRPM4 does not directly interact with those proteins; 2) the assembly of TRPM4-copine 3/vinculin/cofilin constitute low abundance complexes, which we are not able to detect by immunoblot; and 3) TRPM4-copine 3/vinculin/cofilin complex formation is dynamically regulated. Despite the lack of coimmunoprecipitation with these FA proteins, it remains possible that the specific localization of TRPM4 at FAs may allow it to serve as a scaffolding protein that underlies the subcellular organization crucial to the efficient dynamic modulation of the activities of these effectors. As such, both the ion channel activity, and protein-protein interactions mediated by the extensive intracellular domains of TRPM4, could be required for defining the role of TRPM4 in cellular migration and contractility. Further studies will reveal the specific nature and role of the diverse protein-protein interactions that define the novel non-conventional mechanisms related to TRPM4 function in these processes.

Our *in vivo* experiments suggest a possible role for TRPM4 during wound closure. Moreover, pharmacological inhibition of TRPM4 performed in zebrafish and mouse models suggests the participation of this channel in early reparative stages, in which cellular migration is critical [60]. Interestingly, several chemical and physical cues that have been reported as TRPM4 modulators play an important role in the regulation of the cellular migration and contractility and are also present in the wound. For instance, changes in [Ca^2+^]_i_ play a major role during wound repair [61]. In addition, TRPM4 modulators, such as H_2_O_2_, PKC, and PIP_2_ [9,62–64] are crucial for integrin-based signaling [65]. PKC is one of the first proteins identified as a component of the FAs and its activity regulates the formation of FAs in fibroblasts [66]. Also, local increases of PIP_2_ in FAs are involved in the regulation of the signaling in the complexes and FA-induced actin polymerization [65,67]. Conversely, an important role of oxidative species during wound healing and the establishment of gradients of H_2_O_2_ across the wound have been demonstrated [68–70]. Therefore, it could be possible that during tissue repair events, fast increases in [Ca^2+^]_i_, coordinated with the H_2_O_2_ gradients, PIP_2_ increases, PKC activity and the mechanical forces occurring in the wound could enhance TRPM4 channel activity. We speculate that this increased TRPM4 activity leads to increased cellular migration and fibroblast contractility, contributing to the repair of the wound. Thus, the concerted activation of TRPM4 could be required for proper and coordinated turnover of the FAs and then, controlling the actin cytoskeleton dynamics by regulating the Ca^2+^ increases occurring during wound repair. These mechanisms could be involved in the regulation of the migratory behavior of the different cell lineages involved in the process and/or mechanical tension generated in the wound during the healing events, contributing to the closure and repair of the wound and/or fibrotic complications. Conversely, TRPM4 activity has been related to different human pathologies, in which cytoskeletal rearrangement might play a major role. Recently, the role of TRPM4 in endothelial-mesenchyme transdifferentiation has been shown [71]. Gerzanich *et al*. (2009) demonstrated that *de novo* expression of TRPM4 predisposes cells to oncotic cell death in spinal cord injury [72] and the participation of this channel in H_2_O_2_-induced cell death during ischemia/reperfusion episodes have also been described [62,73]. Moreover, increases in TRPM4 mRNA have been observed in prostate cancer [74–76], in B-cell non-Hodgkin lymphoma and in a human cervical-uterine tumor samples and cervical-uterine cancer derived cell lines [77,78]. Together with the data presented here, these studies suggest a role of TRPM4 in mechanotransduction in both physiological and pathophysiological conditions. The participation of TRPM4 in these processes deserves further investigation.

## Supporting Information

S1 Checklist ARRIVE Checklist. (DOCX)

S1 Fig Anti-TRPM4 (TA1008, Origene) antibody validation. Immunoreactivity of the antibody in COS-7 cells expressing TRPM4 protein by immunoblotting and immunofluorescence assays COS-7 cells transfected with a TRPM4 FLAG-tagged plasmid. A. Immunoblot from COS-7 cells lysates transfected with pcDNA4/TO (MOCK) and pcDNA4/TO-FLAG-TRPM4. Membranes were incubated with mouse mAb anti-TRPM4 (TA1008, Origene; right) and then, stripped and reprobe with rabbit pAb anti-FLAG (F7425, Sigma; left). B. Immunofluorescence staining of COS-7 transfected with pcDNA4/TO-FLAG-TRPM4. Cells were stained with Hoechst (blue) mouse mAb anti-TRPM4 (green) and rabbit pAb anti-FLAG (red).
(TIF)

S2 Fig TRPM4 is expressed in FAs of skin fibroblasts in *ex vivo* cultures. Immunofluorescence staining of fibroblasts from mouse skin grafts. Cells were stained with Hoechst (blue), mouse mAb anti-TRPM4 (green), Actin-stain 555 phalloidin (magenta). Scale bar correspond to 5 μm.
(TIF)

S3 Fig Spatiotemporal expression of TRPM4 gene in zebrafish skin. Lateral view (anterior left, posterior right, dorsal up and ventral down) of 24, 36, 48, 60 and 72 hpf embryos. In situ hybridization for TRPM4b (upper panels) and TRPM4c (bottom panels) gene expression since 24 to 72 hpf in the skin of the tail are shown. Arrowheads indicate expression in the skin. Neuromasts (nm), notochord (nt), pronephros (p) and cloaca (c) are indicated. Scale bar: 1 mm.
(TIF)

S4 Fig TRPM4 does not coimmunoprecipitate with vinculin, copine 3 and cofilin. Immunoprecipitation of heterologous TRPM4 from a plasma membrane- enriched protein fraction from HEK293 cells transfected with pcDNA4/TO and pcDNA4/TO-FLAG-TRPM4 (see Materials and methods for details). Immunoblot of input (I), immunoprecipitation products (IP) and flow-through (FT) from this immunoprecipitation assay are showed. Open arrowhead shows the band corresponding to copine 3 protein, black arrowhead shows IgG(H) from anti-FLAG antibody. Asterisk corresponds to an undetermined band.
(TIF)

S1 Movie Focal adhesion dynamics in shRNA^Scramble^ cells. MEFs cells cotransfected with shRNA^Scramble^ and EGFP-Paxillin, serum-starved and stimulated with 5%v/v FBS in HBSS. Images were recorded for 30 min every 1 min. Scale bar: 10 μm.
(AVI)

S2 Movie Focal adhesion dynamics in shRNA^TRPM4^ cells. MEFs cells cotransfected with shRNA^TRPM4^ and EGFP-Paxillin, serum-starved and stimulated with 5%v/v FBS in HBSS. Images were recorded for 30 min every 1 min. Scale bar: 10 μm.
(AVI)

S3 Movie Wound closure in zebrafish larvae (Untreated). Seventy six hours post fecundation (hpf) embryos were anesthetized (0.003% w/v tricaine), mounted in 1% w/v low melting agarose filled acrylic chambers and the tail was wounded with a 0.125 mm–diameter dissection needle. *In vivo* images of wound closure were recorded for 30 min every 2 min.
(AVI)

S4 Movie Wound closure in DMSO-treated zebrafish larvae. Seventy-two hpf embryos were treated with 0.1% v/v DMSO at 28°C in E3 medium for 4 h. Then, the embryos (76 hpf) were anesthetized (0.003% w/v tricaine), mounted and the tail was wounded with a 0.125 mm–diameter dissection needle. *In vivo* images of wound closure were recorded for 30 min every 2 min.
(AVI)

S5 Movie Wound closure in 9-phenanthrol-treated zebrafish. Seventy-two hpf embryos were treated with 20 μM 9-phenanthrol at 28°C in E3 medium for 4 h. Then, the embryos (76 hpf) were anesthetized, mounted and the tail was wounded with a 0.125 mm–diameter dissection needle. *In vivo* images of wound closure were recorded for 30 min every 2 min.
(AVI)
