# Supplementary figures and images for: TRPM4 Is a Novel Component of the Adhesome Required for Focal Adhesion Disassembly, Migration and Contractility
Source: PLoS One. 2015 Jun 25;10(6):e0130540. doi: 10.1371/journal.pone.0130540 (PMC4482413; doi:10.1371/journal.pone.0130540)

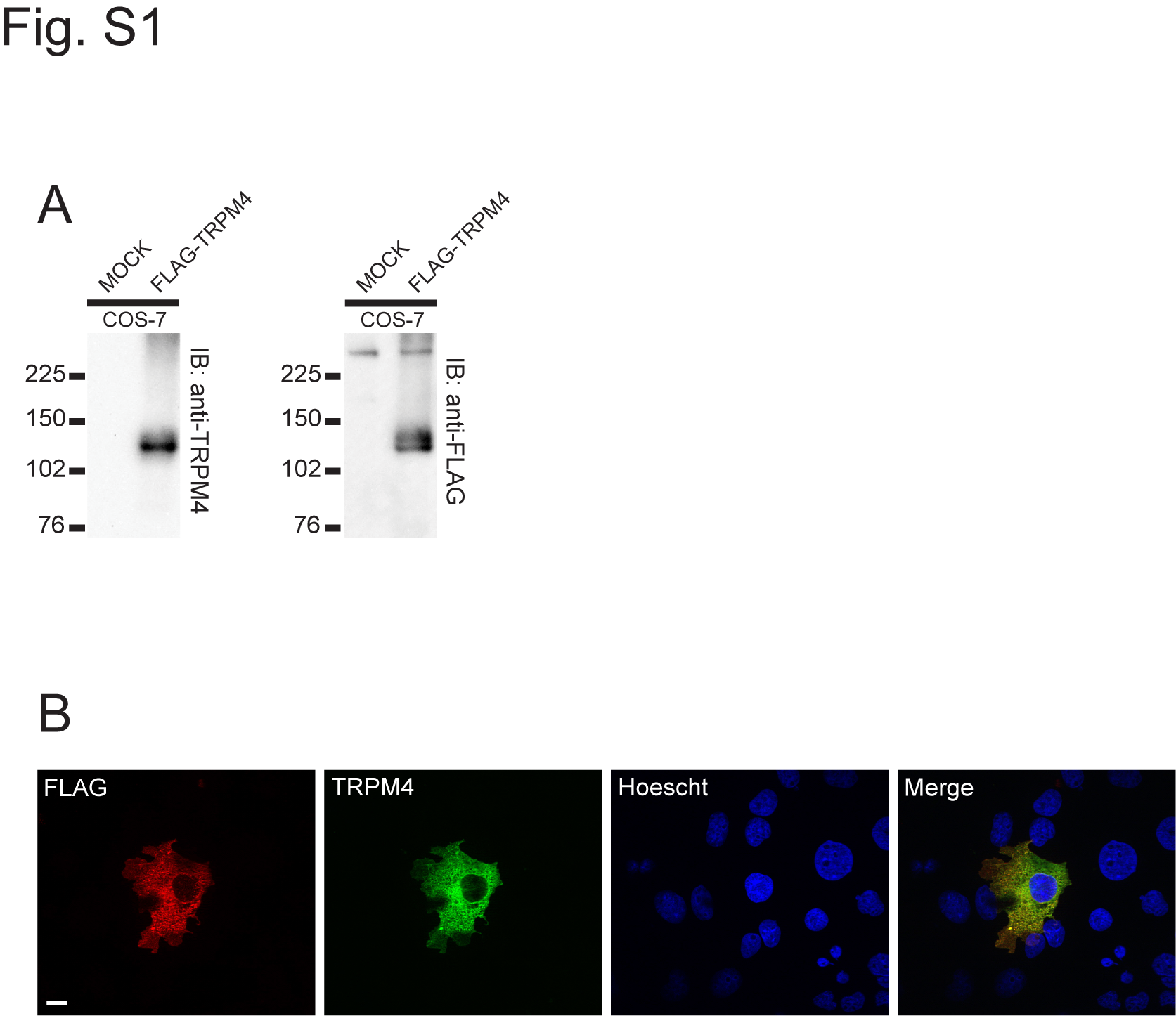

Supplement: S1 Fig — Immunoreactivity of the antibody in COS-7 cells expressing TRPM4 protein by immunoblotting and immunofluorescence assays COS-7 cells transfected with a TRPM4 FLAG-tagged plasmid. A. Immunoblot from COS-7 cells lysates transfected with pcDNA4/TO (MOCK) and pcDNA4/TO-FLAG-TRPM4. Membranes were incubated with mouse mAb anti-TRPM4 (TA1008, Origene; right) and then, stripped and reprobe with rabbit pAb anti-FLAG (F7425, Sigma; left). B. Immunofluorescence staining of COS-7 transfected with pcDNA4/TO-FLAG-TRPM4. Cells were stained with Hoechst (blue) mouse mAb anti-TRPM4 (green) and rabbit pAb anti-FLAG (red). (TIF) [file pone.0130540.s002.tif]

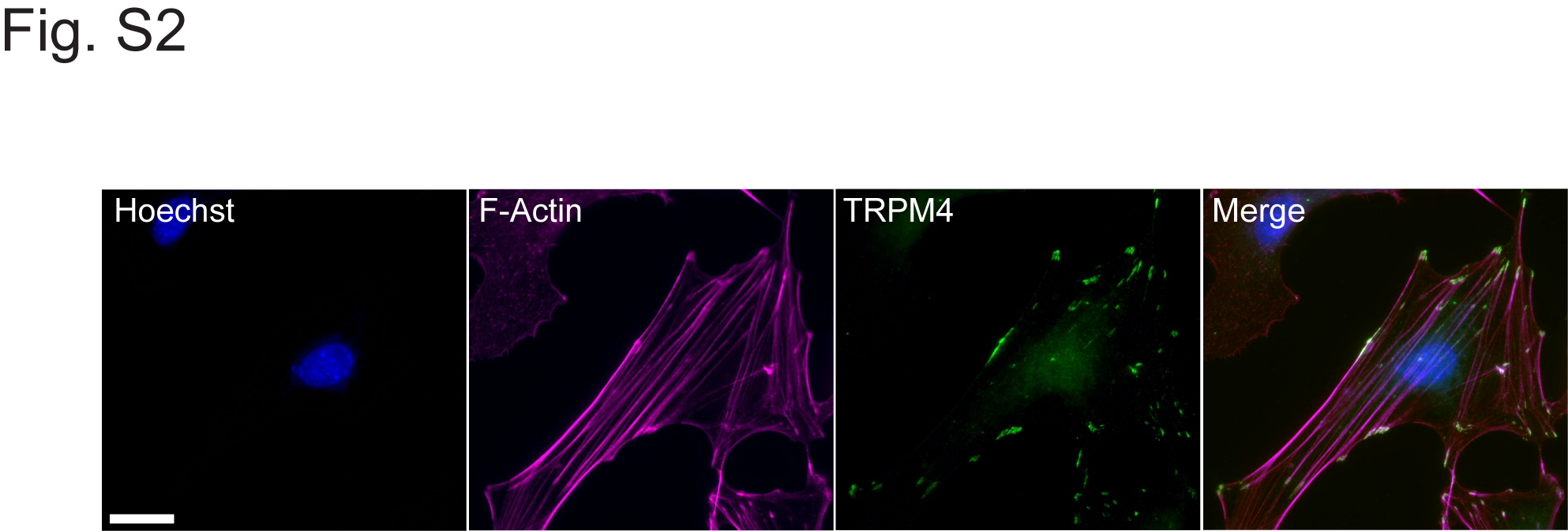

Supplement: S2 Fig — Immunofluorescence staining of fibroblasts from mouse skin grafts. Cells were stained with Hoechst (blue), mouse mAb anti-TRPM4 (green), Actin-stain 555 phalloidin (magenta). Scale bar correspond to 5 μm. (TIF) [file pone.0130540.s003.tif]

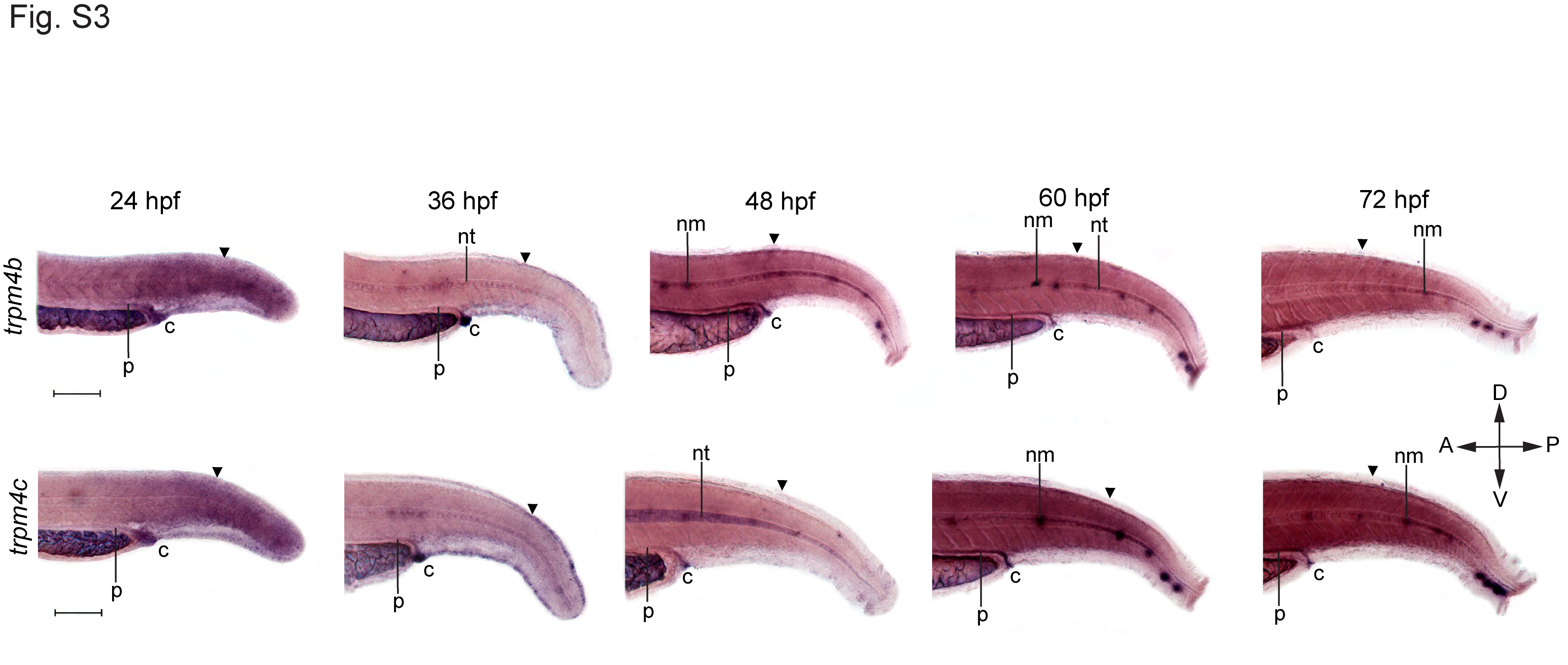

Supplement: S3 Fig — Lateral view (anterior left, posterior right, dorsal up and ventral down) of 24, 36, 48, 60 and 72 hpf embryos. In situ hybridization for TRPM4b (upper panels) and TRPM4c (bottom panels) gene expression since 24 to 72 hpf in the skin of the tail are shown. Arrowheads indicate expression in the skin. Neuromasts (nm), notochord (nt), pronephros (p) and cloaca (c) are indicated. Scale bar: 1 mm. (TIF) [file pone.0130540.s004.tif]

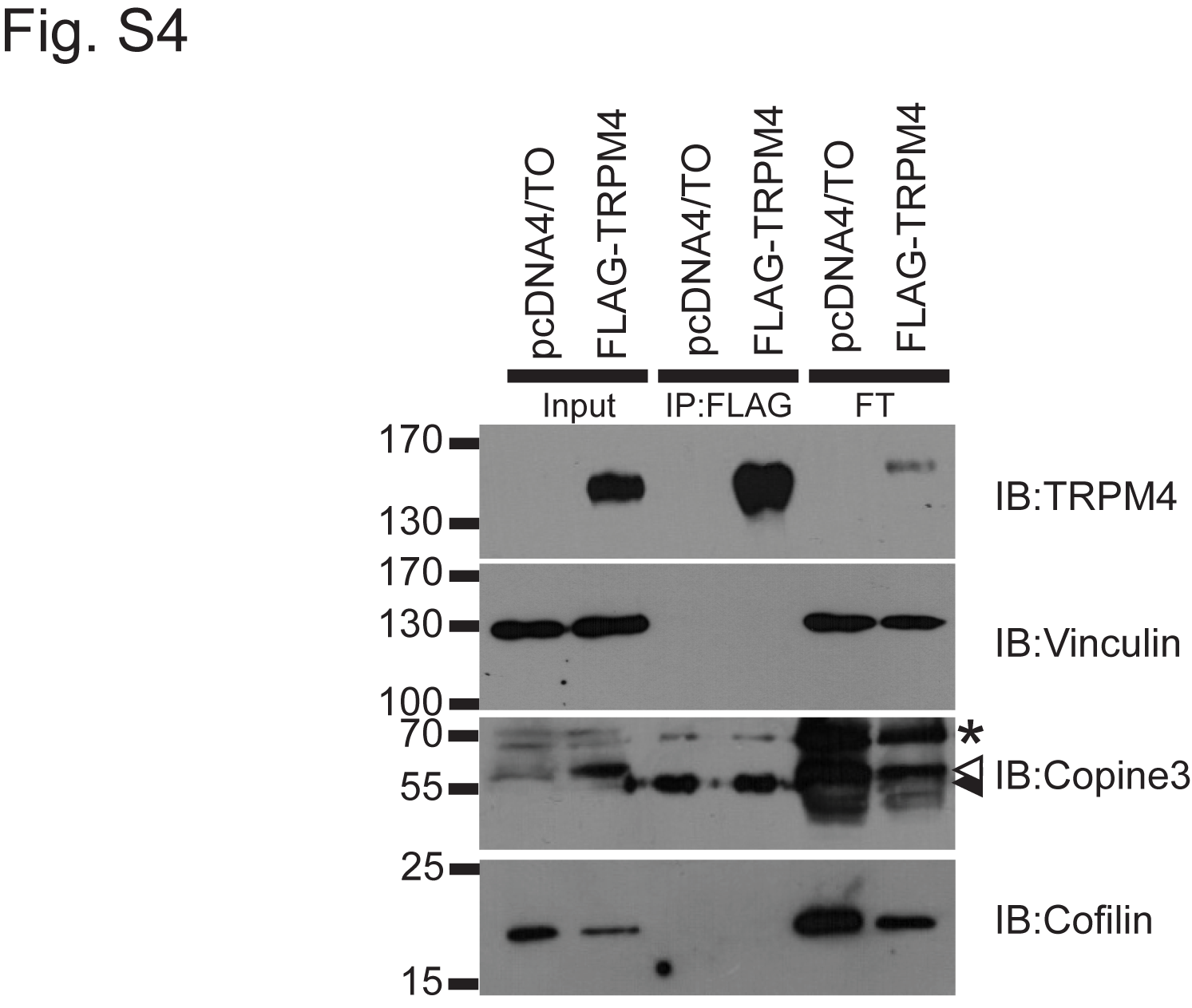

Supplement: S4 Fig — Immunoprecipitation of heterologous TRPM4 from a plasma membrane- enriched protein fraction from HEK293 cells transfected with pcDNA4/TO and pcDNA4/TO-FLAG-TRPM4 (see Materials and methods for details). Immunoblot of input (I), immunoprecipitation products (IP) and flow-through (FT) from this immunoprecipitation assay are showed. Open arrowhead shows the band corresponding to copine 3 protein, black arrowhead shows IgG(H) from anti-FLAG antibody. Asterisk corresponds to an undetermined band. (TIF) [file pone.0130540.s005.tif]
